# The Suppression of Taboo Word Spoonerisms Is Associated With Altered Medial Frontal Negativity: An ERP Study

**DOI:** 10.3389/fnhum.2020.00368

**Published:** 2020-09-04

**Authors:** Tobias A. Wagner-Altendorf, Carolin Gottschlich, Carina Robert, Anna Cirkel, Marcus Heldmann, Thomas F. Münte

**Affiliations:** ^1^Department of Neurology, University of Lübeck, Lübeck, Germany; ^2^Institute of Psychology II, University of Lübeck, Lübeck, Germany

**Keywords:** taboo word utterance, coprolalia, spoonerisms of laboratory induced predisposition paradigm, medial frontal negativity, N450, conflict, inhibition

## Abstract

The constant internal monitoring of speech is a crucial feature to ensure the fairly error-free process of speech production. It has been argued that internal speech monitoring takes place through detection of conflict between different response options or “speech plans.” Speech errors are thought to occur because two (or more) competing speech plans become activated, and the speaker is unable to inhibit the erroneous plan(s) prior to vocalization. A prime example for a speech plan that has to be suppressed is the involuntary utterance of a taboo word. The present study seeks to examine the suppression of involuntary taboo word utterances. We used the “Spoonerisms of Laboratory Induced Predisposition” (SLIP) paradigm to elicit two competing speech plans, one being correct and one embodying either a taboo word or a non-taboo word spoonerism. Behavioral data showed that inadequate speech plans generally were effectively suppressed, although more effectively in the taboo word spoonerism condition. Event-related potential (ERP) analysis revealed a broad medial frontal negativity (MFN) after the target word pair presentation, interpreted as reflecting conflict detection and resolution to suppress the inadequate speech plan. The MFN was found to be more pronounced in the taboo word spoonerism compared to the neutral word spoonerism condition, indicative of a higher level of conflict when subjects suppressed the involuntary utterance of taboo words.

## Introduction

The constant internal monitoring of speech is a crucial feature of human cognition and the basis for the rapid, seemingly effortless and fairly error-free process of our speech production.

One way to try model internal speech monitoring is the perceptual loop theory: The monitor uses the speech perception system for trouble detection, and then initiates the processes of interruption and repair in parallel (Levelt, 1983; Hartsuiker and Kolk, 2001; Hartsuiker, 2014). The perceptual loop theory is an elegant account of speech monitoring because it models speech monitoring as listening to oneself and thus assumes no specialized device or mechanism for error detection.

More recently, however, it has been argued that internal speech monitoring takes place through detection of conflict between response options, which is subsequently resolved by a domain general executive center, localized in the anterior cingulate cortex (ACC) (Nozari et al., 2011; Gauvin et al., 2016). The conflict-based error-detection model of speech production can e.g., account for the dissociation between comprehension and error-detection ability observed in aphasic patients, which the perceptual loop theory cannot (Nozari et al., 2011). Speech monitoring has thus been linked to conflict monitoring (Nozari et al., 2011; Gauvin et al., 2016), as reflected e.g., in the error-related negativity (ERN) and the N2 and medial frontal negativity (MFN) components, and it has been speculated that conflict between representations of intended and actual speech may be a reliable method for detecting speech errors (Yeung et al., 2004; Nozari et al., 2011). Speech errors are thought to occur because two (or more) competing speech plans become activated, and the subject is unable to inhibit the erroneous plan(s) prior to vocalization (Möller et al., 2007).

Inadequate speech plans thus have to be suppressed. This in particular is essential, if the articulation of the erroneous speech plan would be socially objectionable, e.g., in the case of the (involuntary) utterance of taboo words or phrases. Severens et al. (2011) refer to former Spanish Prime Minister José Zapatero’s speech in a 2011 press conference, where he blended the words “favorecer” (to favor) and “apoyar” (to support) into “follar” (which is a vulgar term for cohabitation). The importance of the adequate suppression of (involuntary) taboo word utterance is supported by the finding that subjects intercept more taboo than neutral errors (Motley et al., 1981; Dhooge and Hartsuiker, 2011).

Besides speech errors due to phonological slips as in the case of Zapatero’s, the rather involuntary utterance of taboo words or phrases can occur as cursing or swearing in (emotionally charged) healthy individuals, while it may also appear as a pathological symptom, like coprolalia in Tourette’s syndrome.

The present study addresses the topic of involuntary taboo word utterance in healthy individuals. One way to try to elicit involuntary speech is the “Spoonerisms of Laboratory Induced Predisposition” (SLIP) paradigm (Motley and Baars, 1976; Möller et al., 2007). Spoonerisms are intended or unintended speech errors, where the initial phonemes of two words are exchanged – named after W.A. Spooner, former dean of Oxford, who coined some of the famous examples, such as “Three cheers for our queer old dean” instead of “Three cheers for our dear old queen.” In the SLIP paradigm, “inductor” word pairs are visually presented, with the task to silently read the words. Every few trials (at regular or irregular intervals), a “target” word pair is presented, which has to be articulated overtly. Spoonerisms are sought to be induced by a phonological make-up, in which the initial phonemes of the inductor and the target word pairs are inverse.

Here, we use the SLIP paradigm in an EEG study to examine the involuntary utterance of taboo words, or the suppression of the involuntary taboo word utterance, respectively – that is, the detection of conflict between two competing speech plans (i.e., between two competing representations of articulatory gestures) and the suppression of the inadequate speech plan (Möller et al., 2007). We use the term “conflict” here in the sense used by conflict monitoring theories, i.e., to mean the coactivation of mutually incompatible responses (Yeung et al., 2004), whereby in the context of the present study we are concerned with preresponse (poststimulus) conflict. Thus, one could say that adequate conflict monitoring – i.e., monitoring of the conflict between the two competing speech plans – is the prerequisite for the inhibition or suppression of the inadequate speech plan, and that (adequate) conflict resolution therefore consists in the suppression of the inadequate plan.

The aim of our study thus is to shed further light on the psychological and neural underpinnings of taboo word utterance and taboo word suppression. As response control is impaired in patients with specific conditions who therefore have problems with involuntary taboo word utterance (e.g., in Tourette’s syndrome, in some types of aphasia, after brain injury) our study has potential relevance for clinical research, too.

Studies investigating the electrophysiological correlates of taboo word suppression up to now are rare. While Möller et al. (2007) report an EEG study on neutral spoonerisms using a SLIP paradigm similar to ours, Severens et al. (2011) report on internal taboo correction using a paradigm with a different and more complex composition of stimulus material, finding a broadly distributed negativity at around 600 ms after the speech prompt, pronounced in the taboo-eliciting condition.

For our study, we hypothesized to elicit a component reflecting conflict between the correct and the “spoonerized” speech plan. Several stimulus-locked event-related potential (ERP) components have been linked to both conflict and language processing, e.g., the N2 and MFN – also referred to as N450 – components (West et al., 2005; West and Bailey, 2012; Larson et al., 2014; Nieuwland, 2019).

We hypothesized the conflict between the competing speech plans to be larger in the potentially taboo word-eliciting condition, reflected thus in a larger conflict-indexing component as compared to the potentially neutral word-eliciting condition (Severens et al., 2011). As it is inappropriate to speak taboo words (particularly in an experimental setting), they have to be carefully monitored; and as taboo word monitoring has been shown to interfere with speech production (White et al., 2017), one can assume that taboo word suppression increases conflict level. Anatomical and functional studies have proven a tight interplay between the ACC and e.g., the amygdala, which speaks for a role of emotional saliency (such as taboo word monitoring) for conflict processing within the ACC (Toyoda et al., 2011).

Behavioral expectations for our experiment were that more neutral word spoonerisms than taboo word spoonerisms would occur, as described in previous studies (Motley et al., 1981; Dhooge and Hartsuiker, 2011; but e.g., Severens et al., 2011, found more taboo than non-taboo errors).

## Materials and Methods

### Participants

A total of 32 healthy participants (24 female, 8 male), aged 18–31 years took part in the study. All participants were right-handed, with normal or corrected-to-normal vision, and German native speakers. All participants were students of the University of Lübeck. Before taking part in this study, participants signed an informed consent form. The study was approved by the local ethics committee.

### Procedure

**FIGURE 1:**
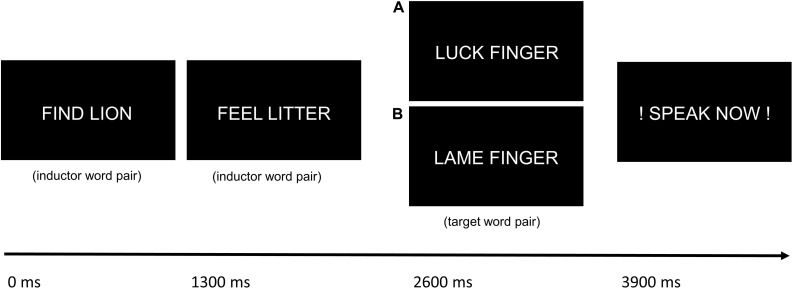
Schematic drawing of the trial procedure with the taboo word spoonerism **(A)** and the neutral word spoonerism **(B)** eliciting condition. After the presentation of 1–4 inductor word pairs, a target word pair with inverse initials was presented, followed by the prompt to overtly articulate the word pair.

German word pairs were presented to the participants for 1200 ms in white against a black background on a video monitor. Interstimulus intervals were 1300 ms. After 1, 2, 3 or 4 “inductor” word pairs (with the same combination of initial letters) that were silently read, one “target” word pair was presented, followed by the prompt to overtly articulate the word pair (“speak now!”). As the target word pair could be presented after a differing amount of inductor word pairs, participants could not predict whether the next trial would be a naming trial. The next inductor word pair was presented after 2300 ms. Target word pairs had inversed initials compared to inductor word pairs, to provoke spoonerisms (e.g., “find lion” - > “feel litter” - > “luck finger” could induce the taboo spoonerism “fuck linger;” see Figure 1). The control condition consisted in potentially spoonerism-inducing word pairs, too, but with neutral content (e.g., “find lion” - > “feel litter” - > “lame finger”). 38 different combinations of taboo-inducing and 38 different combinations of non-taboo-inducing target word pairs were used, so that one session comprised a total of 152 target word pairs (after 1, 2, 3 or 4 inductor word pairs) in each condition. 152 different inductor word pairs were used. Neutral and taboo-eliciting stimuli were controlled for similar length and lexical characteristics [so that the initial and final letters were the same and the words also belonged to the same part of speech (adjective, verb, noun); e.g., “weiche” and “weiße” as inductors to elicit the spoonerisms “scheiche” (neutral) and “scheiße” (taboo)]. Word stimuli for both conditions were selected for high frequency in everyday language. Target word pairs in the taboo and in the neutral word spoonerism condition were equally common in everyday language, as indicated by the absence of a significant difference in mean google results for the target words in both conditions (mean taboo: 450,325,287; mean control: 356,133,958; *p* = 0.75, unpaired *t*-test). Also, a comparison of the target word pairs in both conditions using the scientific database SUBTLEX-DE (Brysbaert et al., 2011) showed no difference in lexical frequency of the stimuli as indicated by lgSUBTLEX value (mean taboo: 2.08; mean control: 2.17; *p* = 0.66, unpaired *t*-test). Moreover, a comparison of the target word stimuli listed on the Berlin Affective Word List Reloaded (BAWL-R; Võ et al., 2009) revealed no difference in emotional valence and arousal between conditions (emotional valence: mean taboo: 0.25; mean control: 0.21; *p* = 0.88; arousal: mean taboo: 2.63; mean control: 2.68; *p* = 0.78; unpaired *t*-tests).

See appendix for a complete list of word stimuli used.

### Data Acquisition

The participants’ vocalizations were digitally recorded using a Rhøde podcast microphone and Audacity^®^ software. Audio files were offline checked for the occurrence of spoonerisms.

EEG was recorded continuously from 30 unipolar tin electrodes placed according to the International 10–20 system, using an electrode cap (Electro-Cap) and a 32-channel Brainamp MR amplifier. Sampling rate was 1 kHz. Band-pass ranged from 0.016 Hz to 1 kHz. A notch filter at 50 Hz was used. Electrode impedances were kept under 5 kΩ. Electrode locations were Fp1/2, Fpz, F3/4, C3/4, P3/4, O1/2, F7/8, T7/8, P7/8, Cz, Fz, Pz, FC1/2, CP1/2, PO3/4, FC5/6, CP5/6. Reference electrode was put on the left earlobe. Vertical (vEOG) and horizontal (hEOG) electrooculograms were monitored from electrodes placed below and above the eye, and at the left and right outer canthi, respectively.

EEG and EOG data were recorded with Acquire^®^ software. For EEG analysis, EEGLAB (Delorme and Makeig, 2004) and ERPLAB (Lopez-Calderon and Luck, 2014) were used. EEG was segmented into 1600 ms intervals (400 ms before, 1200 ms after reference point). To remove ocular artifacts, ICA (independent component analysis) was used. To account for non-ocular artifacts such as amplifier blocking or sudden jumps in amplitude the “moving window peak-to-peak threshold” function was used, with a threshold potential individually adjusted for each participant after visual inspection of long stretches of EEG. Epochs containing these artifacts were excluded from the analysis. Seven participants were excluded from analysis, due to a high artifact rate. ERPs were filtered with a 20 Hz low-pass filter. For baseline correction, baseline was defined as the interval from -100 to 0 ms. From the resulting data, averages for each segment and participant were determined, and subsequently, grand averages were calculated across all participants.

### Statistical Analysis of ERP Data

Visual inspection of the grand average ERP waveforms revealed a broad late negative component between ca. 500 and 1000 ms after the presentation of the target word pair. We used repeated measures ANOVAs to test for differences between the taboo word spoonerism and the neutral word spoonerism condition in the ERPs. Visual inspection revealed that the taboo/neutral word condition differed most between 600 and 800 ms. Electrodes F3/Fz/F4/C3/Cz/C4 were analyzed. For the mean amplitude of the time window 600–800 ms we calculated an ANOVA comprising the repeated measures factors condition (taboo word spoonerism vs. neutral word spoonerism) anterior/posterior (frontal vs. central) and hemisphere (left vs. central vs. right). *Post-hoc t*-tests were calculated to reveal which electrode site drove the interaction.

To test for possible N2 differences after target word pair presentation, the mean amplitude in the interval between 280 and 380 ms was analyzed, and for post speech prompt analysis, the mean amplitude in the interval between 400 and 600 ms was analyzed, because visual inspection revealed possible differences between conditions at these time windows.

On mean, 12.6% (9.28 out of 73.64) (taboo condition) and 12.5% (9.12 out of 72.72) (control condition) of trials were discarded due to high artifact rate, for target word pair-locked analysis. 24.4% (18 out of 73.64) (taboo condition) and 24.2% (17.6 out of 72.68) (control condition) of trials were discarded due to high artifact rate, for speech prompt-locked analysis.

Only trials, where the target word pair appeared on position 3 or 4 (not on position 2 or 5) were analyzed. This was done to ensure adequate priming by at least two inductor word pairs (exclusion of position 2), and to prevent predictability of target word pair occurrence and speech prompt (exclusion of position 5).

Statistical analysis was done with R^®^ 3.6.1 (Lawrence, 2016; R Core Team, 2019). For ERP data analysis, repeated measures ANOVA with Greenhouse-Geisser correction was performed. Uncorrected F, but corrected *p*-values are reported.

### Statistical Analysis of Behavioral Data

Behavioral data (participants’ qualitative vocalizations) was analyzed for the occurrence of taboo word and neutral word spoonerisms. Statistical analysis (Wilcoxon signed rank test) was done with R^®^ 3.6.1.

## Results

### Behavioral Data

On average, each participant produced 2.03 spoonerisms overall, i.e., in both conditions (in 2 × 152 target word pairs; i.e., 0.67%). More spoonerisms occurred in the neutral word (1.38/0.91%) than in the taboo word condition (0.65/0.43%). Wilcoxon signed rank test revealed statistical significance (V = 6; *p* = 0.017).

### ERP Data

**FIGURE 2:**
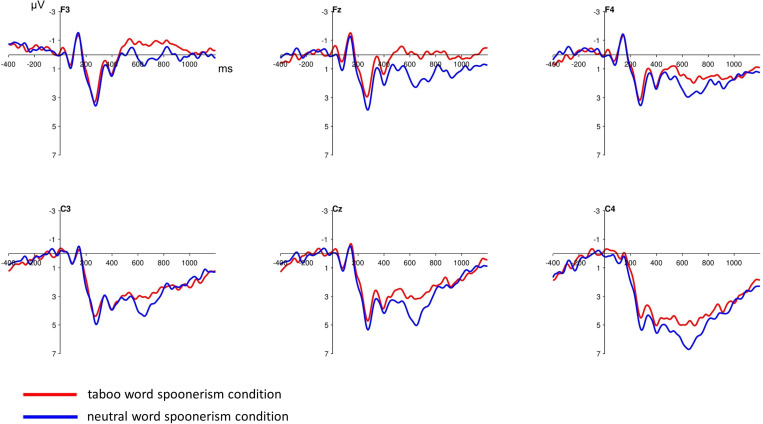
Stimulus-locked grand average ERP waveforms at frontocentral electrodes after the presentation of the target word pair. The broad negative component is more pronounced when a taboo word spoonerism is suppressed, indicative of a pronounced conflict resolution process in this condition. Baseline used is -100 to 0 ms. The displayed waveforms were filtered with a 20 Hz low-pass filter.

**FIGURE 3:**
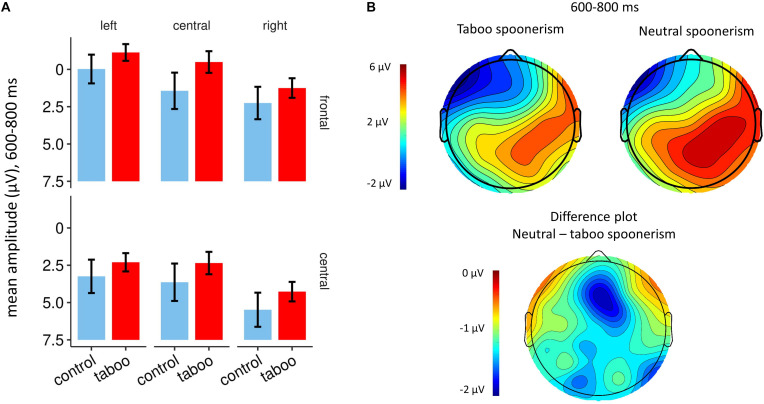
**(A)** Bar plots showing mean amplitudes at 600–800 ms after presentation of the target word pair, for F3, Fz, F4, C3, Cz, and C4 electrodes. The taboo word spoonerism condition is depicted in red, the neutral (control) word spoonerism condition is depicted in blue. **(B)** Topography of the taboo word spoonerism and the neutral word spoonerism condition (top), and of the difference waveform between the two (bottom), at 600–800 ms after the presentation of the target word pair, showing the frontocentral localization of the component.

The inspection of the ERPs revealed a broad frontal negative component, beginning at ca. 500 ms after the target word pair presentation, that was more pronounced in the taboo word spoonerism condition (see Figures 2, 3). The difference between the two conditions in negativity was greatest at 600–800 ms. Analyzing the frontocentral electrodes (F3, Fz, F4, C3, Cz, C4), we performed a 2 (condition: taboo vs. neutral word condition) × 2 (anterior/posterior: frontal vs. central) × 3 (hemisphere: left vs. central vs. right) repeated measures ANOVA with Huynh-Feldt correction, which revealed a significant condition^∗^anterior/posterior^∗^hemisphere interaction (*F* = 4.97; *p* = 0.013). Anterior/posterior and hemisphere main effects were highly significant (*F* = 57.27; *p* < 0.001 and *F* = 17.03; *p* < 0.001, respectively), condition main effect was not significant (*F* = 3.05; *p* = 0.094). *Post-hoc t*-tests for the taboo vs. neutral word spoonerism condition for single electrodes revealed a significant condition main effect for Fz electrode (*p* = 0.03); for other electrode sites no significant condition main effect was found.

Additionally, the N2 component was analyzed in the time window between 280 and 380 ms, with a 2 (condition: taboo vs. neutral word condition) × 2 (anterior/posterior: frontal vs. central) × 3 (hemisphere: left vs. central vs. right) repeated measures ANOVA, for frontocentral electrodes, showing no significant condition main effect (*F* = 1.88; *p* = 0.183), and no significant condition^∗^anterior/posterior^∗^hemisphere interaction (*F* = 2.3; *p* = 0.124).

**FIGURE 4:**
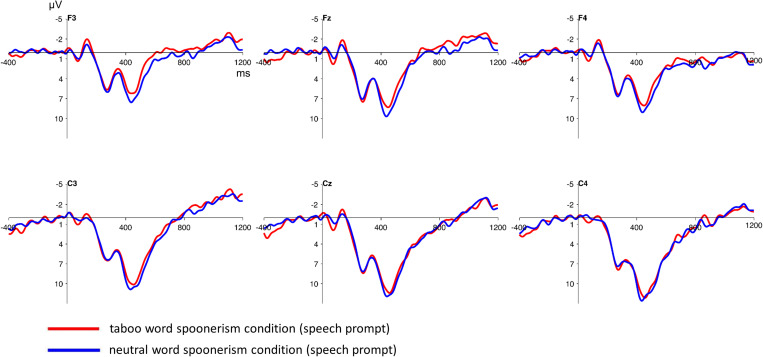
Stimulus-locked grand average ERP waveforms at frontocentral electrodes after the presentation of the speech prompt/pronunciation cue. No significant differences between the taboo word spoonerism and the neutral word spoonerism condition are present. Baseline used is -100 to 0 ms. The displayed waveforms were filtered with a 20 Hz low-pass filter.

The inspection of the ERPs following the speech prompt showed no significant differences between the taboo word spoonerism and the neutral word spoonerism condition (see Figure 4). This was statistically verified using a 2 (condition: taboo vs. neutral word condition) × 2 (anterior/posterior: frontal vs. central) × 3 (hemisphere: left vs. central vs. right) repeated measures ANOVA, for frontocentral electrodes, showing no significant condition main effect (*F* = 1.81; *p* = 0.191), and no significant condition^∗^anterior/posterior^∗^hemisphere interaction (*F* = 1.7; *p* = 0.2) in the time interval between 400 and 600 ms.

## Discussion

The present study investigated the occurrence and suppression of taboo word spoonerisms in a SLIP task. Only very few actual (overtly spoken) spoonerisms occurred, which is in line with previous work on laboratory induced spoonerisms (e.g., Severens et al., 2011, found about 1% spoonerisms; Möller et al., 2007, found 10% full and partial spoonerisms, but prescreened the participants for their inclination to produce spoonerisms). However, non-taboo word spoonerisms occurred twice as often as taboo word spoonerisms. This speaks for a tendency to intercept more taboo than neutral errors, consistent with previous studies (Motley et al., 1981; Dhooge and Hartsuiker, 2011).

ERP analysis revealed a frontocentral negativity component following the presentation of the target word pair (i.e., the potentially spoonerism-inducing and thus conflict-inducing word pair). This component can be identified with the medial frontal negativity (MFN), which has been implicated in conflict detection and resolution (Larson et al., 2014), and is thus likely to reflect a conflict between two competing speech plans – i.e., between the correct speech plan and the “spoonerized” speech plan insinuated by the phonological make-up of the inductor and the target word pairs.

Previous studies, using e.g., Stroop tasks, have shown that conflict detection and resolution go along with a pronounced N450 or MFN component over frontal and parietal electrodes (Liotti et al., 2000; West, 2003; Xiang et al., 2013; Chouiter et al., 2014; Larson et al., 2014; Rey-Mermet et al., 2019). We prefer the term MFN here, since the timing of the negative deflection varies across studies and can extend beyond 700 + ms, eventually reflecting two or more transient ERP effects (see, e.g., West and Bailey, 2012). The neural generator of this component is thought to be the anterior cingulate cortex (ACC) (West, 2003). The inhibition of speech errors in particular, both taboo and neutral word errors, has been localized to the right inferior frontal gyrus (Severens et al., 2012).

In the response conflict literature, another – response-locked – ERP component has been extensively discussed: the error-related negativity (ERN). The ERN is a frontocentral negative-going deflection which occurs after the subject has made an erroneous response (Falkenstein et al., 1990; Gehring et al., 1993). It is thought to monitor conflict procession when a response conflict occurs (when a task concurrently activates more than one response tendency) (Ridderinkhof et al., 2004; Larson et al., 2014). Yeung et al. (2004) and Yeung and Cohen (2006) propose, within the conflict response model, that the ERN reflects postresponse conflict between the executed, erroneous response and the competing, correcting response tendency.

As stimulus-locked ERP components associated with conflict, such as the N2 and the MFN, and the response-locked ERN component show a similar topography and have both been localized to the ACC, it has been suggested that the same process underlies both peaks and that the (caudal) ACC thus serves as a central monitor of both stimulus induced and response conflict (Veen and Carter, 2002; Botvinick et al., 2004; Trewartha and Phillips, 2013). One might thus argue that this central conflict monitor becomes active both after a (conflict-inducing) stimulus – e.g., in the case of two competing speech plans –, and after an (erroneous) response. Whereas the postresponse potential – the ERN – is relatively short and circumscribed, the poststimulus potential – the MFN – shows a rather broad temporal distribution. The competing speech plans activated by the SLIP paradigm are thus likely to induce a conflict during response selection driven by e.g., caudal ACC activity (Botvinick et al., 2001, 2004) that is reflected in ERP components ERN and MFN.

In our study, we found, statistically significant for Fz, the MFN to be more pronounced in the taboo word spoonerism condition, i.e., when a taboo word was suppressed, compared to the suppression of a non-taboo word spoonerism. If the MFN is related to self-monitoring and reflects conflict detection and resolution, this thus indicates that the suppression of a taboo word spoonerism is associated with a higher level of conflict – especially in a social context, where the utterance of taboo words is inappropriate, e.g., in a scientific experiment at a university (for the correlation of the likelihood of saying a taboo word, e.g., as a swear word, and the social context, see Jay, 2000). As the ERP after the speech prompt shows no difference between the taboo vs. neutral spoonerism condition, it is unlikely that the observed difference is due to lexical differences between the conditions.

This is consistent with a finding by Severens et al. (2011), who, in a SLIP task, observed a broadly distributed negative wave at around 600 ms that was pronounced in the taboo-eliciting condition. However, that difference was found after the speech prompt/pronunciation cue, not after the target word pair presentation (after the target word pair presentation, no significant difference between conditions was present). In contrast, Möller et al. (2007), using a (non-taboo eliciting) SLIP task, found a negativity between 400 and 600 ms time locked to the target word pair presentation. The negativity was pronounced when participants actually produced a spoonerism, and thus interpreted as reflecting conflict between two competing speech plans. This is in line with our finding of taboo word suppression reflecting conflict that was time locked to the target word pair presentation, too (with no observable difference after the speech prompt). A possible explanation for the differences in the observed ERP between Möller et al. (2007) and Severens et al. (2011), as well as our study, might be the different composition of stimulus lists. Due to the higher amount of word pairs that did not require pronunciation and additional “filler” word pairs in the stimulus list used by Severens et al. (2011), ERP differences between the taboo word and the non-taboo word spoonerism condition might have shifted to the period after the presentation of the pronunciation cue.

The suppression of the (taboo word) spoonerism requires the inhibition of the “spoonerized” speech plan, i.e., the erroneous response. Response inhibition, and the performance in stop-signal tasks in particular, have been modeled as a “race” between a go process and a stop process (Verbruggen and Logan, 2008). The response is inhibited, when the stop process finishes before the go process. The maxim to behave socially appropriate, i.e., not to utter taboo words in a context such as a scientific experiment, thus might embody the internalized stop signal which, in the case that the “spoonerized” speech plan contains a taboo word, triggers the stop process and consecutively inhibits the erroneous response. This interpretation is supported by an fMRI study by Severens et al. (2012) showing that the inhibition of taboo words, as well as the inhibition of socially neutral words, is associated with activation of the right inferior frontal gyrus (rIFG). As the rIFG has been linked to externally triggered inhibition (e.g., Xue et al., 2008), Severens et al. (2012) conclude that external social rules (“do not utter taboo words!”) become internalized and act as stop signal.

It has to be mentioned that, as the present study focused on (the inhibition of) involuntary taboo word utterance, one might wonder whether participants realized after some trials that the study design seeks to elicit taboo word utterances, and thus the words would, partially, loose their taboo character. However, the overt articulation of a taboo word (as a mistake when prompted to pronounce a neutral word) is still very objectionable, especially in a context such as a scientific experiment. Another possible limitation is the transfer of the observed taboo word suppression in healthy individuals to the taboo word suppression or utterance under pathological conditions, e.g., in coprolalia in Tourette’s syndrome. One might question that strictly the same pathophysiological mechanisms underlie both the rather involuntary utterance of taboo words e.g., in a SLIP task and in coprolalic tics in patients. Further research regarding differences and similarities between the two is needed, e.g., by characterizing the brain mechanisms involved in suppressing involuntary taboo word utterance, as observed in the SLIP task, in Tourette patients.

To conclude, the present study focused on the inhibition of the involuntary utterance of taboo words, i.e., on the interception or suppression of an inadequate (“taboo-spoonerized”) speech plan in a SLIP task. The inhibition of the inadequate speech plan was found to be more pronounced in the taboo word spoonerism condition compared to the control (i.e., non-taboo word spoonerism) condition. This, first, is reflected in our behavioral data – taboo errors were intercepted twice as often as neutral errors. Second, it is reflected in our electrophysiological findings – taboo word-suppressing trials were associated with a pronounced MFN component indicative of higher conflict detection and resolution compared to neutral word-suppressing trials. Further studies are needed, in particular investigating not only stimulus-locked, but also response-locked ERP components, such as the ERN, to taboo word utterance and suppression.

## Data Availability Statement

The raw data supporting the conclusions of this article will be made available by the authors, without undue reservation, to any qualified researcher.

## Ethics Statement

The studies involving human participants were reviewed and approved by the Ethikkommission der Universität zu Lübeck, Ratzeburger Allee 160, 23538 Lübeck. The patients/participants provided their written informed consent to participate in this study.

## Author Contributions

TW-A, CG, MH, and TM contributed to the design and conductance of the study and analysis of data. TW-A wrote the first draft of the manuscript. CG, CR, AC, MH, and TM contributed to critical revision of the manuscript. All authors contributed to the article and approved the submitted version.

## Conflict of Interest

The authors declare that the research was conducted in the absence of any commercial or financial relationships that could be construed as a potential conflict of interest.
